# Smartphone Prospects in Bridge Structural Health Monitoring, a Literature Review

**DOI:** 10.3390/s24113287

**Published:** 2024-05-21

**Authors:** Ekin Ozer, Rolands Kromanis

**Affiliations:** 1 School of Civil Engineering, University College Dublin, D04V1W8 Dublin, Ireland; 2 Department of Civil Engineering and Management, University of Twente, 7522 NB Enschede, The Netherlands; r.kromanis@utwente.nl

**Keywords:** structural health monitoring, dynamic identification, damage detection, modal analysis, computer vision, vision-based sensing, sensor technologies, mobile sensing, smartphones, bridge dynamics, signal processing, level of mobility (LoM)

## Abstract

Bridges are critical components of transportation networks, and their conditions have effects on societal well-being, the economy, and the environment. Automation needs in inspections and maintenance have made structural health monitoring (SHM) systems a key research pillar to assess bridge safety/health. The last decade brought a boom in innovative bridge SHM applications with the rise in next-generation smart and mobile technologies. A key advancement within this direction is smartphones with their sensory usage as SHM devices. This focused review reports recent advances in bridge SHM backed by smartphone sensor technologies and provides case studies on bridge SHM applications. The review includes model-based and data-driven SHM prospects utilizing smartphones as the sensing and acquisition portal and conveys three distinct messages in terms of the technological domain and level of mobility: (i) vibration-based dynamic identification and damage-detection approaches; (ii) deformation and condition monitoring empowered by computer vision-based measurement capabilities; (iii) drive-by or pedestrianized bridge monitoring approaches, and miscellaneous SHM applications with unconventional/emerging technological features and new research domains. The review is intended to bring together bridge engineering, SHM, and sensor technology audiences with decade-long multidisciplinary experience observed within the smartphone-based SHM theme and presents exemplary cases referring to a variety of levels of mobility.

## 1. Introduction

The growth of the world population requires our civil infrastructure to expand, thus keeping our society functioning and resilient in times associated with the aftermath of the COVID-19 pandemic, climate change, and energy transition. Bridges are crucial components of civil infrastructure. They provide links to locations separated by obstacles. Their continuous and safe operation is essential. Recent failures of bridges, may they be coming close to their design life (such as the Morandi Bridge [1,2,3]) or recently built (such as Tretten Bridge [4]), are a harsh message to their owners, and for management and maintenance teams, there is a strong need for keeping bridges safe to use and fit for their purposes. This can be achieved by implementing structural health monitoring (SHM).

SHM has developed as a computational and sensor-technology-driven research field with a gaining pace in the last four decades [5,6]. SHM can be performed at local and global levels, i.e., global and local structure behavior [7,8]. SHM at the local level, in general, can be regarded as a localized inspection, in which, for example, cracks or concrete delamination are quantified. SHM at the global level collects and analyzes data related to the overall/global performance of the structure, for example, the load and response mechanism and vibration parameters. Due to the automation needs in condition assessments and, if any, damage detection, SHM plays a promising role in the management and efficient operations of infrastructure assets. SHM has a rich history of development with numerous benchmark reviews and keynote contributions [9,10,11,12,13,14,15,16], many of which highlighted today’s technological challenges in advance. SHM, unified through Rytter’s framework [17], recently was boosted due to the level of smartness and mobility [18] as the information technology foundations led to the next industrial revolution [19].

However, the SHM domain still has yet to reach its full potential due to the advanced labor and instrumentation needs. Thrive in mobile sensing and digital transformation is about to propose a new SHM frontier, ubiquitous sensing where community-led sensor networks scan the conditions of infrastructure assets [20]. Smartphones constitute one giant source of infrastructure data, and in the last decade, we have witnessed the rapid expansion of smartphone-based bridge-monitoring applications. While most of the research is still at a proof-of-concept stage, the application scales have started increasing, and the technology readiness level gradually maturates.

In this review, we provide the recent advances in smartphone technologies and their applications in the field of SHM with a particular emphasis on bridge infrastructures. The key feature in bridge infrastructure is that large signal-to-noise ratios offer mechanics-based information about the structural states of bridges, despite the limited fidelity of smartphone sensors. While there is a variety of classification means to formulate and cluster the progress in smartphone-based bridge monitoring, we observe three distinct development paths geared towards (i) vibration-based approaches using invasive techniques, e.g., accelerometers [21], (ii) vision-based and noncontact approaches based on non-contact cameras and deformation monitoring [22], and drive-by sensing approaches making use of smartphones embedded in vehicles [23]. Besides, new concepts in bridge-health monitoring offer transitionary cases, which take new forms of mobility, those focusing on emerging SHM problems, e.g., large-scale network implementations [24] and unconventional sensory usage [25] and emerging digital paradigms [26].

Accordingly, the review is structured as follows: Section 2 introduces a smartphone with its built-in sensor technologies. Section 3 reports the state-of-the-art invasive smartphone-based bridge vibration monitoring. Section 4 covers the state-of-the-art computer vision-based (CV-based) applications used for bridge SHM enabled by smartphone cameras. Section 5 presents a transitionary case, which is neither remote nor dedicated, i.e., drive-by sensing. Section 6 presents two case studies that exemplify the notion of the Level of Mobility (LoM) introduced in the paper. Section 7 addresses emerging matters that are in the process of development and highlights outstanding problems in smartphone-based SHM and new paradigms that are likely to impact the next decade’s SHM agenda not only limited to smartphone usage. The final section, Section 8, gives a summary, conclusions, and future directions.

## 2. Smartphone Technology and Bridge SHM

Before projecting a comprehensive review on different research tracks in smartphone-based bridge SHM, it is vital to express how the literature evolved within the last decade. The first iOS-compliant smartphone, iPhone, was released in 2007; see a history of smartphone industrial advances in [27]. Soon after, in the early 2010s, the first smartphone applications making use of embedded accelerometers for a geoscience purpose were introduced, such as iSeismometer [28] and iShake [29]. Embedded triaxial accelerometers in smartphones could function as seismometers and eventually form a dense seismic network [30]. Since seismometers sense the earth’s motion following seismic activity, the same principle could apply to other deformable or vibratory physical media, i.e., civil infrastructure. Accordingly, the core condition assessment discipline SHM was introduced to the smartphone sensing concept, where the first journal papers provided the early proof [31,32].

**Figure 1:**
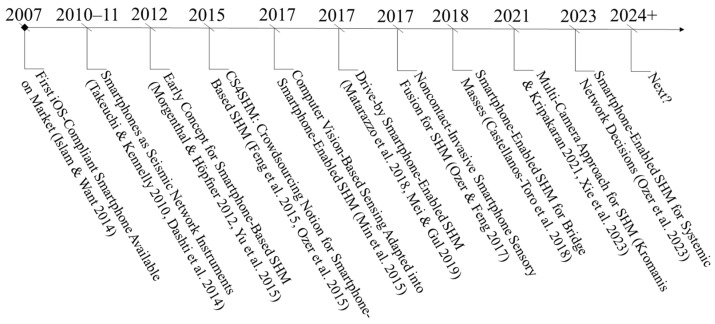
A timeline of smartphone evolution from its advent to complex and large-scale SHM research (2007 to 2024), examples above refer to in [27,28,29,31,32,33,34,35,36,37,38,39,40,42], respectively.

The mid-2010s witnessed a new SHM paradigm making use of smartphone-user data, i.e., crowdsourcing-based SHM [33,34]. Also, in the mid-2010s, computer vision applications using smartphones were initiated for deformation measurements [35]. These were followed by vehicular innovation in SHM, alternatively, drive-by SHM, where smartphone data are collected from moving vehicles to characterize bridge health [36,37]. Smartphone sensing features increased in complexity, and sensor fusion strategies merged invasive (accelerometer) techniques with remote and noncontact (vision-based displacement) techniques [38]. Apart from multiple techniques integrated into each other, different studies merged multiple smartphone camera data for an enhanced deformation monitoring experience [39,40]. Complexity was not limited to the alternative physical meaning of different sensor technologies but also the scale of the SHM problem, e.g., bridge populations [41], bridge networks, and high-level analytics, such as decision-making [42]. Figure 1 presents the timeline of these key progresses in smartphone-based SHM.

Above, we mentioned the advent of crowdsourcing in SHM applications of bridges. While most of the smartphone research falls into the proof-of-concept of a crowdsourcing-based bridge monitoring approach, few of them explicitly express the notion of crowdsourcing and citizen-induced uncertainties, and a few others present an actual crowdsourcing dataset rather than controlled verification scenarios. The authors recommend a classification criterion for crowdsourcing-based SHM: when dedicated researchers or their controlled task-performers are assigned to the tests, our suggestion is to classify the effort as a proof-of-concept crowdsourcing-based SHM rather than a direct claim of crowdsourcing. While we understand that the level of control can be interpreted subjectively, herein, a few examples of controlled task-performers are (i) researchers/interns involved in the research of concern, (ii) researchers volunteered while having the knowledge of the technical domain, (iii) closely controlled participants with detailed supervision of each crowdsourcing step and the tests themselves, and further examples can be derived within a similar context concerning the task-performance knowledge and instructional levels.

**Figure 2:**
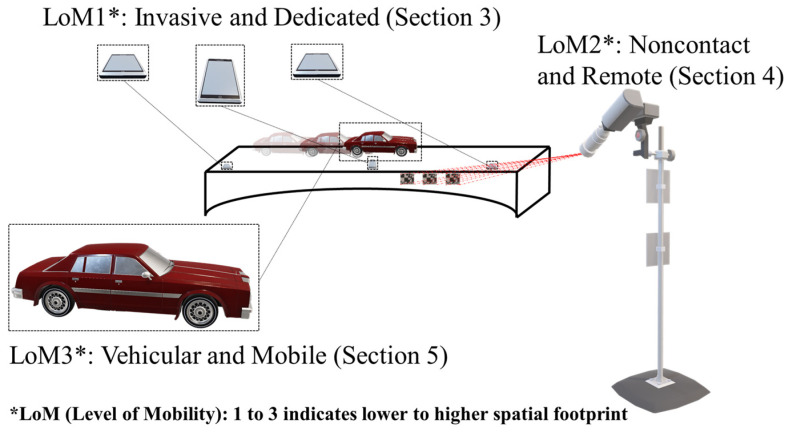
Level of mobilities (LoMs) observed on a smartphone-engaged bridge monitoring ecosystem.

**Figure 3:**
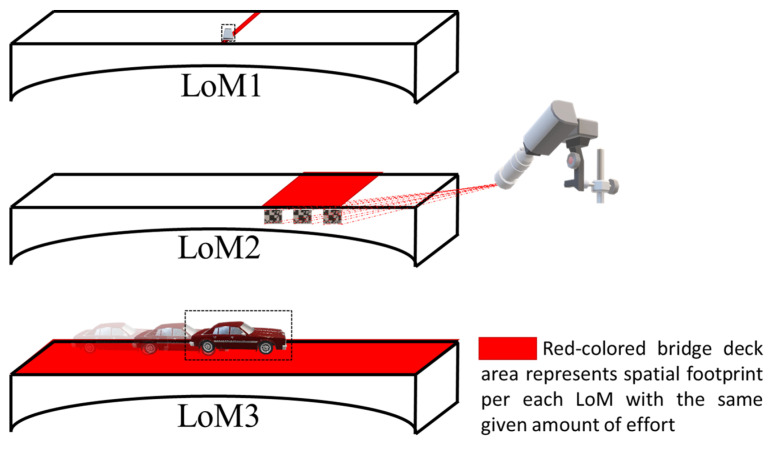
Spatial footprint comparison for LoMs.

Parallel with the key advances expressed above, in the forthcoming sections, the advances in smartphone-based SHM, with a particular emphasis on bridge infrastructure are categorized into three segments according to the Level of Mobility (LoM): LoM1, invasive smartphone sensing via dedicated sensor positioning; LoM2, computer vision-based smartphone sensing via remote and noncontact monitoring; and LoM3, drive-by smartphone sensing via indirect vehicular monitoring. Accordingly, Section 3, Section 4 and Section 5 address LoM1, LoM2, and LoM3, respectively. Figure 2 conceptually presents LoMs, and Figure 3 indicates their spatial reach. LoMs and their key characteristics are summarized in Table 1.

**Figure 4:**
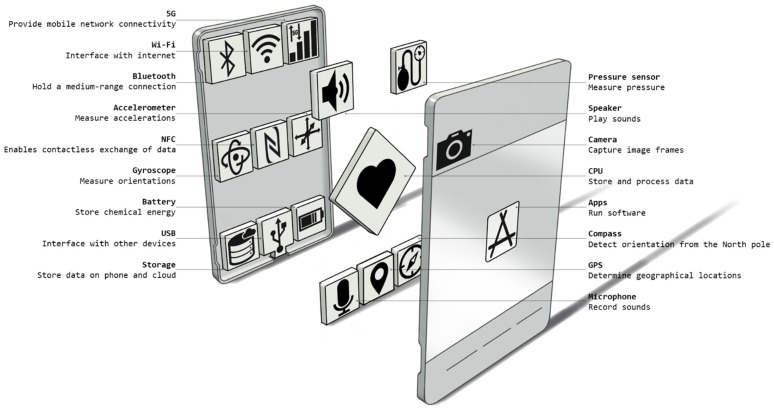
Conceptual breakdown of smartphone components.

As a recap of the smartphones’ technological potential in vibration monitoring, the broad availability and low cost of smartphones justify the recent investment in their use for the SHM of bridges. Smartphones contain built-in sensors, storage capabilities, processors, and broadband communication networks, such as the cellular 4/5G network, Wi-Fi, and Bluetooth. Built-in sensors include an accelerometer, camera, microphone, gyroscope, magnetometer, Global Positioning System (GPS), and Light Detection and Ranging (LiDAR). A visual illustration of typical smartphone components with their description is given in Figure 4. While each of these technologies can serve a purpose, the authors would like to avoid repetition since various smartphone studies have introduced such basic information, and it is considered common knowledge that can be found in the literature [20,43,44].

Similarly, a summary of the smartphone-related sensor technology and its application in SHM is conducted in references [20,43,44], and their amplified roles in next-generation SHM through data sciences is further discussed in [45,46]. This type of technology is relevant to standard condition monitoring applications, as it can be used either as SHM systems or as nondestructive testing during bridge inspections with the usage of various embedded sensors with different measurement contexts. It is important to note that the following content’s scientific focus is on bridge infrastructures, rather than an exhaustive list of SHM applications with a broad infrastructure description. Therefore, this review is dedicated to providing a novel formulation that distinguishes the mobility differences among data collection scenarios, denoted as LoM. In that sense, there are significant differences in terms of how the structural response is sampled in the joint context of spatiotemporal domains, and LoM terminology will hopefully contribute to the evolution of smartphone-based SHM research by separating the three technological pillars. In the following sections, the authors classify the advances in smartphone-based SHM with a special focus on bridges, based on the LoM principles introduced in this section.

## 3. Invasive Smartphone Sensing for Bridge Monitoring

Applications of invasive smartphone sensing were predominantly pursued by two core groups at the Dalian University of Technology and Columbia University with a rising global interest from many other contributions from all around the world. Invasive smartphone-based SHM is a transition between traditional sensing protocols for bridge monitoring and emerging paradigms, such as the mobile and drive-by sensing techniques. This section focuses on LoM1, whereas Section 5 will explain how it transitions into LoM3.

### 3.1. Cable Force Measurement and Invasive Bridge Monitoring Applications in Dalian Group

Concerning the Dalian group, the first journal paper published on smartphone-based SHM refers to 2012 [31], whereas similar exercises were presented in conferences in the same year [47]. An evolved version of this initial validation was presented in [32], where the authors demonstrate an initial validation of a mobile SHM method that utilizes smartphones [48]. They looked at the acceleration signals received from smartphones placed on bridge cables, used them to identify frequency content, and compared them with a traditional instrument, i.e., an accelerometer. With this validation study, they have shown that tension cable force can be inferred from a smartphone providing a low-cost alternative to traditional and expensive instrumentation and eventually expanded the validation protocol to an actual cable-stayed bridge study. The same researchers also presented a framework for the cloud operation nature of smartphones for cable vibration monitoring with an app called Orion-CC [49] and discussed the potential of using it in a public participation framework [50]. They performed similar observations with a broader range of experimental and field studies, where many cables were monitored with smartphone sensors and the frequency–cable force relationships were compared using a reference system [51]. And eventually, they initiated a computer vision-based procedure to use smartphone cameras for the displacement monitoring of bridge cables, discussed in further detail in Section 4 [52].

**Figure 5:**
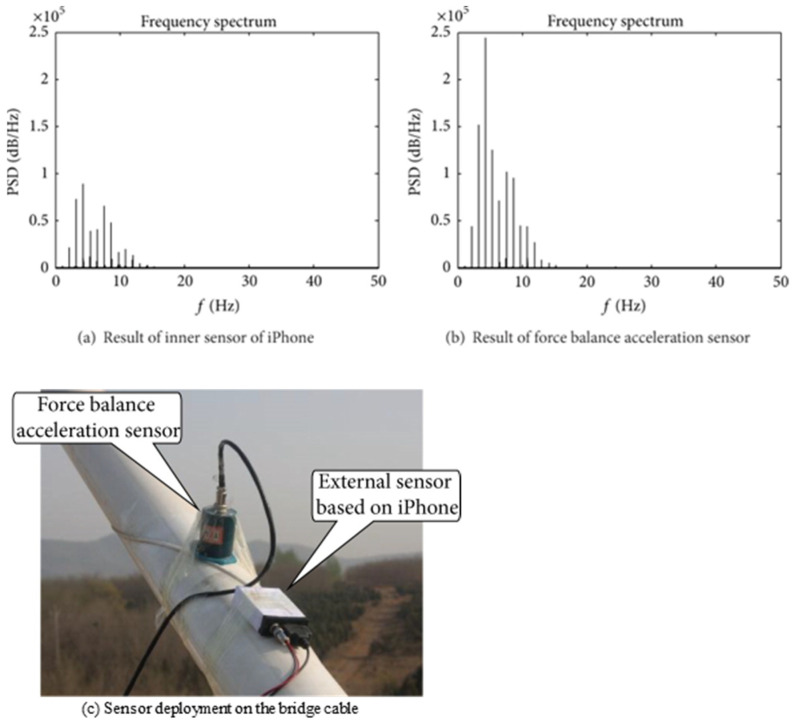
Bridge cable installation of smartphone sensor: smartphone vibration frequency comparisons with the reference data (**a**,**b**) and sensor configuration (**c**), Yu et al. (2015) [32].

The second phase of the Dalian group’s research on SHM studies continued with different technological approaches. They monitored a laboratory-scale bridge’s displacements with a laser projected on a reference plate and the imagery observed by the smartphone, with an app developed for the purpose, D-Viewer [53]. While the smartphone keeps track of the projected laser blob via a camera in a non-contact manner, it still needs to have a dedicated position within the vicinity of the plate. They also identified the hoisting girder problem engaged in the bridge construction process and made use of smartphone sensors to sense extreme orientations [54]. Figure 5 shows an example setup for bridge cable vibration monitoring and frequency spectrum comparisons between the smartphone and a reference sensor, by Yu et al. (2015) [32].

### 3.2. Modal Identification, Model Updating, and Decision Analysis at Columbia Group

Within a similar timeframe, Columbia University developed a research agenda focusing on a preliminary assessment of smartphone sensors for bridge monitoring purposes and citizen-induced uncertainties associated with an uncontrolled sensing environment [21]. The authors identified smartphone sensing as a potential vibration-monitoring and modal identification platform [33] and introduced the idea of crowdsourcing as an emerging bridge-vibration data acquisition protocol [34]. To the authors’ best information, they collected the first crowdsourced bridge vibration data from a pedestrian link bridge and compared the frequency estimations made by a three-layered software platform engaging web and mobile applications (apps) relying on internet connection [34]. The comparisons with reference systems were promising, yet short-time vibration samples have shown a visible dispersion in terms of identification accuracy. To investigate the sources of these uncertainties further, the authors identified three sources of error that can be fixed with smart features of the embedded sensors. These uncertainties were related to (i) spatiotemporal features of uncontrolled instrumentation (e.g., lack of a synchronous system) that can be solved through geolocation detection and energy-to-power conversion [55], (ii) orientation errors caused by directional changes of the smartphone during operation and a remedy via gyroscope/magnetometer input for coordinate system transformation [56], (iii) and biomechanical effects when there is an intermediary mechanical system, e.g., pedestrians, interfering between the bridge and the smartphone to be solved using a personalized transfer function development procedure and cancellation of the pedestrian effects to retrieve bridge vibrations [57]. A summary of these works is presented in [58].

The Columbia group also expanded on the vibration-sensing and modal identification protocol to multi-output and large-scale experimentation with a landmark bridge example, the Golden Gate Bridge, comparing a smartphone-based synchronization-free operational modal analysis approach with a traditionally instrumented frequency domain decomposition process [59]. The authors acquired bridge modal frequencies and mode shapes despite the distributed nature of smartphone clocks. The authors expanded the smartphone clock problem into a probabilistic description and identified a Kernel distribution formulation to represent the smartphone sampling rate imperfections [60]. At the same time, the authors linked modal identification findings to calibrate bridge finite element models [42]. Stemming from the benchmark works developed for a seismic shake table test [61,62], the authors interpreted smartphone-based bridge SHM as a cyber-physical process and utilized model updating for structural reliability supporting decision-making [63]. Eventually, they expanded the model-updating-based decision analysis protocol to a bridge network where they monitored 20 bridges with smartphones [42].

### 3.3. Global Bridge SHM Contributions Supporting the LoM1 Scheme

The studies on invasive smartphone-based bridge SHM are not limited to the groups’ work mentioned above. The first journal article dedicated to smartphone-based SHM is proposed as lab trials of different sensor technologies embedded in smartphones by Weimar, Germany [31]. The same group extended their research in the smartphone-based SHM area with more studies on the cable-force identification [64], smartphone-controlled Raspberry pi sensors [65], and a non-contact modal analysis approach discussed further in Section 7 [66]. Another early example from Europe was [67], which utilized nonlinear acoustics techniques to detect cracks on metal plates using smartphones. While there was no explicit callout for bridges in the study [68], it was another early study summarizing potential the application areas of smartphone sensors, including SHM. While the core notion of smartphones has everyday and non-expert access to it, a few examples looked at the population of bridge-vibration characteristics. Among those, the highest number that is available to the authors’ knowledge is reported in [41].

Sensor fusion was among the trending topics of the late 2010s, i.e., GNSS and accelerometers [69,70] and computer vision and accelerometers [38,71] (some of these examples do not directly refer to bridges, but the techniques introduced therein are transferrable). Other than these [72,73], similar to [34], dedicated applications to acquire vibration data from smartphones in an integrated manner were developed. While [72] performed multi-output modal identification in a synchronous setting, the latter two relied on single-output identification results [34,73]. As can be seen above, the majority of the examples were focused on the identification stage and its representation as damage-sensitive features. A few examples extrapolated identification knowledge onto the calibration of finite element models, such as [74] or [63] and, in some exceptional cases, region-scale bridge models [42]. Not only the structural state but also the serviceability limit state attracted attention within the smartphone community with a focus on civil infrastructure. For example, [75] utilized smartphones to study the serviceability features of a series of structure types, including bridges. One final group with a contribution to invasive techniques was from Saitama, Japan, where the researchers initiated a feasibility study investigating the acquisition performance of smartphones based on a cable-stayed bridge [76] and later on modified the framework for long-term, continuous, and remote measurements obtained from bridges [77]. The same group eventually developed a machine learning classifier distinguishing device bias, earthquakes, traffic, and ambient vibrations from each other based on a permanently installed six-smartphone-accelerometer system [78].

## 4. Computer Vision-Based Smartphone Sensing for Bridge Monitoring

**Figure 6:**
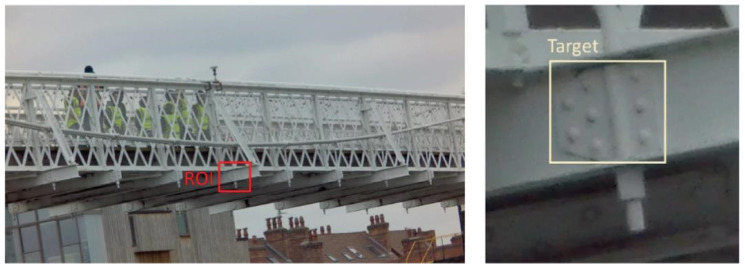
Image frame with an ROI (**left**) and ROI with a target (**right**) [82].

Computer vision-based (CV-based) sensing in SHM involves analyzing digital image(s) carrying out the following tasks: image classification, object detection and tracking, segmentation, and optical flow. A detailed review of CV-based SHM at local and global levels together with the techniques involved in the listed tasks can be found in [8]. Visions and frameworks of using fully CV-based SHM systems for structural identification, in which both the structural input and output are obtained by cameras and CV-based techniques with both conventional cameras and smartphones, can be found in [79,80]. This section reviews CV-based bridge monitoring attempts at global and local levels using smartphones at LoM2. The principle behind the target tracking commonly used in SHM applications is that a target(s) is specified in a set region of interest (ROI), where it is sought in the consecutive image frames. Defining an ROI reduces the computational expenses. Structural displacements are usually small, and therefore, targets are expected to have small movements. Commonly used target tracking techniques are as follows: (1) template matching, where the target is a template usually selected as a rectangular area containing sufficient information characterizing the target, (2) feature point (or keypoint) technique, in which features, such as edges, characterizing a selected target are extracted, (3) optical flow, in which the optical flow of pixels of a target is estimated, and (4) shape-based tracing, where a shape of, usually, an artificial target, is used [81]. Figure 6 (left) shows an image frame from a CV-based measurement system where an ROI is defined. A target is then identified and chosen within the selected ROI (see Figure 6 (right)) [82].

### 4.1. SHM at the Global Level

When considering SHM at the global level, all three types of a structure’s response (i.e., static, dynamic, and quasi-static) can be measured using smartphone cameras. Kromanis et al. [83] demonstrated the capabilities of smartphone cameras capturing consecutive image frames, from which accurate measurements of all three types of structural responses in a laboratory environment were computed. Three image-processing algorithms were used and compared. It was found that measuring the static response and estimating vibration parameters (i.e., dynamic response) can be performed with equally good results using multiple image-processing algorithms and cameras. However, capturing an accurate thermal response, which is driven by slowly changing temperature, was challenging. Studies in which smartphones were used for estimating all types of bridge responses, starting with static, following by dynamic, and finishing with a quasi-static response, are reviewed in the following sub-sections.

#### 4.1.1. Measuring a Static Response

A bridge static response results from applied loads that, in general, are at lower frequencies than bridge natural frequencies. Two examples are the response (i) from traffic on the bridge, e.g., pedestrians, vehicles, trains crossing a bridge [84,85], and (ii) static load testing, such as placing load trucks or other known loads on specific locations on the bridge [86,87]. Studies relevant to such cases are reviewed in this section.

A multiple-camera position [39] or position-independent multi-epoch imaging [88] approach was developed for acquiring comparable response measurements (in the form of vertical displacements) from multiple camera positions in the perspective to the bridge. Blobs (black circles), which were drawn at known locations on the surface of the structure, served as visual targets. Jo et al. [89] developed a rotation-invariant image processing technique to obtain accurate measurements of a laboratory setup and a bridge. The technique allows for the measurement of accurate displacements regardless of the angle between the smartphone camera and the target on the bridge. Martini et al. [90] proposed a fully CV-based method for bridge model updating, in which the aim was to accurately capture displacement influence lines, which can be used for SHM purposes. iPhones were used to capture the structural displacements induced by vehicles crossing the laboratory bridge. For studies by [89,90], predesigned targets were used.

Voordijk and Kromanis [91] collected mid-span displacements of a 27 m-long steel girder bridge subjected to controlled pedestrian crossings with a smartphone and a modified GoPro with a zoom lens. Small static bridge displacements (>1 mm) were not being accurately captured with the smartphone camera, which was installed approximately 15 m from the midspan of the bridge. Derivatives from target displacement, such as inclination angles, curvatures, and strains, can be used for assigning the performance of bridges. Obiechefu et al. [92] demonstrated the feasibility of detecting different levels of damage from measurement histories of vehicle crossings of a laboratory bridge using inclination angles and curvatures. Obiechefu [93] further demonstrated that strains from target displacements can also be estimated to the accuracy needed for damage detection of the laboratory bridge. Tian et al. [94] demonstrated that accurate displacements of a subway passing bridge could be collected with a smartphone camera that was set 60 m away from the passing. From the displacements of three targets, they estimated the deformed shape of the bridge at three states of the subway on the overpass. Studies also include applications of a static load on a fiber optic cable to measure strains using a smartphone camera with a microscope [95,96] and the same as the previous but for both static and dynamic [97].

#### 4.1.2. Estimating Vibration Parameters

When it comes to the laboratory environment, vibration parameters can be accurately estimated, especially vibration frequencies [98,99]. Similar object-tracking algorithms that are used when analyzing images collected with conventional digital cameras are also deployed with smartphones. Some examples are the tracking of a known shape, such as a full circle, keypoing matching, digital image correlation, and optical flow. Ozer et al. [38] obtained the same vibration properties with smartphones attached to (i.e., accelerometer data) and a smartphone focused on (i.e., CV-based data) a laboratory frame model. Alzughaibi et al. [71] proposed a community-based multi-sensory SHM system, in which a smartphone accelerometer and camera are used to detect earthquakes and record measurements during them. The premise is that a smartphone’s camera is facing the ceiling, on which an object serves as a target that is tracked during the earthquake. Zhu et al. [98] developed a marker-free method based on optical flow for estimating vibration parameters. In their study, iPhone 8P was deployed to capture the dominant frequency of a laboratory cable stayed bridge. A smartphone with a zoom lens to measure the dynamics of a shaking table results were a good match with a laser [35] and also by [100].

Some smartphone apps were developed for the purpose of tracking a target on a structure. An example is D-viewer developed by Dalian University of Technology, China. It tracks a blob that is projected on a surface [101]. The app monitors dynamically structural displacements by means of a common laser device and a projection plate. Papers by this research group focus on the development and enhancement of the app [53,101,102] and an application for bridge response measurements [52,94,103]. The limitation of the app is its ability to track a single target in the form of a blob, although multiple clusters of pixels defining a target can be tracked in an image frame. This is also the unique strength of CV-based systems/measurements.

#### 4.1.3. Capturing Bridge Thermal Responses

A bridge thermal (i.e., quasi-static, slowly changing) response is governing bridge long-term displacement. Daily and seasonal bridge responses to temperature variations are known to exceed significantly short-term traffic-induced responses [104]. Currently, the limited applications of CV-based systems for long-term measurement collection are found in the literature. Kromanis et al. [83] demonstrated that the thermal response of a structure can indeed be captured using a smartphone camera in a laboratory environment. Borah [105] measured the thermal response of a laboratory truss subjected to accelerated temperature cycles and a full-scale bridge. Two image-processing techniques, Kanade-Lucas-Tomasi (KLT) feature-tracking and digital image correlation (DIC), were compared. DIC was found to be more robust than the KLT algorithm for thermal response measurements; however, it had higher computational costs. The bridge thermal response (in the form of strain) was measured over a period of 12 h. Although the estimated response resembled ambient temperature measurements of the day and the anticipated thermal response, it could not be confirmed against the ground truth, i.e., measurements with a reliable and tested measurement unit. Bridge bearing longitudinal, vertical, and transverse displacements were estimated for the 24 h monitoring of the Nakano Bridge located at Kawaguchi, Saitama, Japan [106]. Three iPhones were used in the study. They were set at a very close distance (<1 m) to the bearing and focused on an artificial target in the form of a QR code. Currently, the longest application of smartphone cameras was performed by Park et al. [107]. They employed two smartphones for a duration of two weeks for measuring the thermal movements of a bridge at is bearings. Images were taken every 10 min. Measured displacements were only 3.11% different from those obtained with a displacement transducer.

### 4.2. SHM at the Local Level and Other Applications

Similarly to the standard techniques that are adapted in CV-based crack detection, a large library of 11,000 images was used to train a pruned fully convolutional network and edge computing; then, a smartphone was used to capture images and perform the reset [108]. The efflorescence and spalling damage detection of masonry structures was performed using a real-time system in [109]. Ni et al. [110] developed crack detection at a close range. Han et al. [111] proposed using smartphone cameras to obtain visual measurements from the first-person perspective. Images from smartphones fixed to a body can be synchronized with the scene seen by operators. Then, trained convolutional neural networks (CNNs) are used to draw and then extract the contour line of the target component in the images by the key points of hands. An analysis of images collected with smartphones for the detection and classification of defects has been successful using transfer learning and CNNs [112]. Relevant studies, in which smartphone cameras were used for image collection, have demonstrated that structural defects can be detected and classified for a variety of civil structures [109,113]. A case study concerning the process of vibration parameter characterization is provided in the forthcoming sections based on the testbed monitored via multiple technologies, i.e., smartphone [80], as well as GNSS [114,115].

## 5. Drive-by Smartphone Sensing for Bridge Monitoring

**Figure 7:**
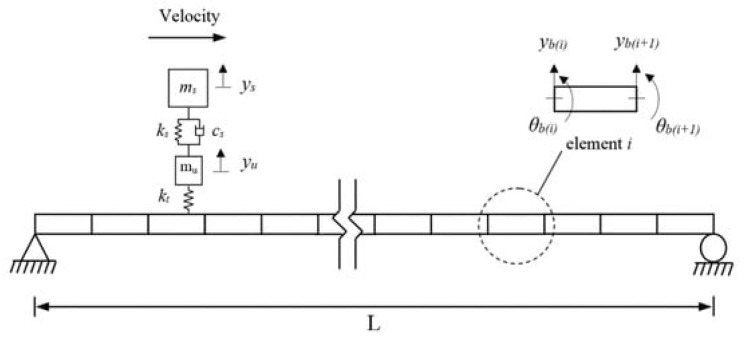
A vehicle–bridge interaction model used for simulating the indirect response, Zhu and Malekjafarian (2019) [120].

Previous sections, Section 3 and Section 4, discussed two LoMs that can be achieved in terms of the spatial footprint of the smartphone-based SHM systems. This section will expand the LoM discussion to a new level (LoM3), with the highest spatial footprint available via fully mobile sensing scenarios. This can be achieved via vehicular sensing processes, which are also the most compatible data-acquisition modes with a crowdsourcing notion. The vehicular approach in the abovementioned smartphone-based monitoring framework is mostly called drive-by SHM [116]. It has also been referred to using different terminologies (e.g., traversing vehicle [117], indirect sensing [118], moving/mobile sensing [119]). Drive-by SHM simply works with the idea of collecting bridge damage sensitive features (e.g., modal parameters) while the instrumented vehicle is in motion. Concerning smartphone-based drive-by SHM, the instrumentation is smartphones and their collaborative equipment in certain cases. Figure 7 shows a representative vehicle–bridge interaction model used to simulate the indirect bridge response in drive-by identification studies [120].

Drive-by SHM using smartphones was introduced in 2017–2018 by a few researchers from Boston, US [36], Belfast, UK [121], and Alberta, Canada [37]. In [36], the authors performed drive-by measurements using a smartphone placed on the dashboard of a vehicle. The authors collected vibration signals for modal frequency estimations using a comprehensive statistical analysis process, arguing that a crowdsensing scenario can improve the identification quality as more data are collected. Likewise, in [37], the authors performed laboratory tests on a small-scale bridge specimen to show the measurement sensitivity to a damaged specimen as a small-scale moving vehicle collects data from a mini bridge. In the case of [121], the authors worked on real testbeds in the Belfast transport network and tagged the spatial footprint of smartphones with external GNSS devices.

More research followed the pioneers above; numerous researchers performed proof-of-concept tests to retrieve modal frequency data from moving vehicles instrumented with smartphones [122,123,124]. Likewise, more advanced techniques were developed, such as an inverse filtering approach for frequency identification [125] and more complex drive-by modal analyses [126] and clock-asynchronous data [127]. Concerning bridges, the drive-by data encapsulates the mechanical features of the bridge, as well as the vehicle, and the interaction with each other. Others incorporated the vehicle–bridge interaction effects for smartphone-based SHM via numerical models [128]. While vehicle–bridge interaction is important for large vehicular excitation sources, e.g., trains [129], smartphone-based sensing solutions can disregard them in certain cases where vehicle effects are minor, e.g., micromobility vehicles [130]. More crowdsourcing research is in progress to link drive-by SHM and smartphone technologies together via modal analysis using moving vehicles [131].

Nevertheless, smartphone-based and drive-by SHM together with crowdsourcing has huge potential to contribute to bridge damage and deterioration assessments, e.g., detecting scour [132]. With the MITICA project, there is a significant investment to expand this concept through densely instrumented realistic testbeds [133]. In realistic environments, it is essential to eliminate the operational effects that can mask damage indicators in modal frequencies [134] and account for the vehicle properties for the precise removal of vehicular effects distorting indirect bridge data [135]. And the next step following drive-by modal identification is damage detection, which can also infer the remaining useful life of beam-like systems [136]. Recent progress in drive-by SHM shows promising progress in the way the contact-point response is representative of modal parameters [137] and accordingly bridge damage [138]. These advancements are reflected in smartphone-based drive-by SHM as well, i.e., modal identification with a small-scale laboratory vehicle [139], and account for complex interactions among micromobility vehicles, pedestrians, and bridges [140]. Finally, it is essential to remind the readers that it is not all about identification/detection accuracy. Drive-by sensing comes with a data-intensive protocol, necessitating reducing the transmission burden to the distributed device networks, which can be achieved with data-compression remedies [141]. More information on the latest advances in drive-by SHM can be found in [142,143].

Having covered all three LoM scenarios, it is essential to provide snapshots of the identification accuracy influenced by different smartphone approaches. One common question in smartphone-related SHM studies is the accuracy of smartphone accelerometers and their product features. The question can be approached in the time domain and in the frequency domain by looking into the signal amplitude and identified frequency content, respectively [33]. Regarding the former, the accuracy in the accelerations strongly depends on the signal-to-noise ratio. Therein, the signal depends on the operational vibrations of the bridge, and the noise is predominantly characterized by the sensor make and model, which is different among smartphone generations and brands. Some earlier studies showed that the accuracy of the amplitude has radically improved within the first few generations of smartphones (i.e., 2008 to 2013) [34]. Moreover, bridges have a higher level of operational vibrations under an ambient environment in comparison to buildings; therefore, they have a relatively good signal-to-noise ratio in numerous cases.

A good signal-to-noise ratio also enables vibration frequencies to be identifiable, reducing the noise floor to a distinguishable level. However, in the frequency domain, there are further issues that require consideration, such as the clock imperfections of smartphones. Earlier studies omitted accounting for clock discrepancies, whereas the later studies improved identification accuracy that could be subjected to drifts due to sampling rate errors. To reflect on these matters with references from the literature, Table 2 presents a set of frequency identification cases with and without clock correction principles. It can be seen that the frequency identification accuracy is within the range of single digits in all reported observations; however, clock corrections can improve this further since target sampling rates and achieved sampling rates are different, constantly observed in the literature, and taken into account in certain examples (e.g., [37,38,59,73,77,131]).

This clock deviation can be simply a deviation from the target sampling rate or can contain more complex features, e.g., a multi-modal distribution along the frequency domain [60]. Other factors influencing the frequency identification accuracy include the driving/riding speed of a vehicle in the case of LoM3, e.g., [130], the loading type, e.g., [33], and the duration of the vibration samples [59]. While LoM1 and LoM3 are influenced by sampling rate errors, such issues have not been reported in computer-vision applications forming LoM2 [83], as far as discovered by the authors. Short-duration samples, indirect media, and speed effects possess combined difficulties towards achieving successful identification results in LoM3 as opposed to LoM1. Another aspect that should be considered is the environmental and operational variability, which can serve as a masking feature if identification results are used as a comparative basis for damage detection.

## 6. Example Case Studies

The sections above attempt to provide a broader picture of the advancements in smartphone-based bridge monitoring and propose the LoM formulation to characterize the fundamental streams of research developed in the last decade. This section is developed to show further details of example smartphone-based bridge monitoring exercises, and two case studies are presented herein: (I) bridge monitoring with smartphone computer vision (LoM2) and (II) bridge monitoring with smartphone accelerometers (LoM1 and somewhat LoM3).

### 6.1. Estimating Bridge Vibration Parameters: A Case Study Utilizing Computer Vision

**Figure 8:**
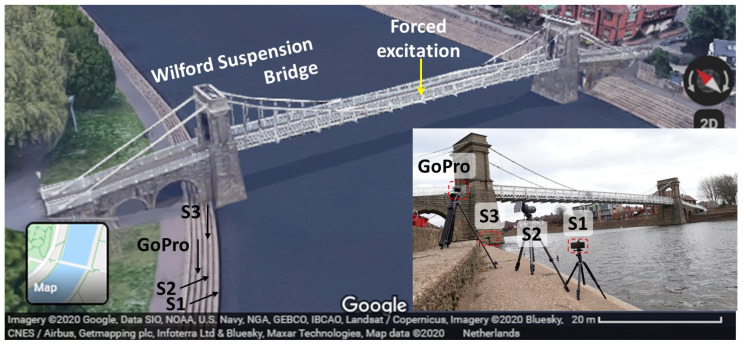
CV-based system for measurement collection of the Wilford Suspension bridge.

The Wilford Suspension Bridge is a 69 m-long pedestrian and aqueduct bridge crossing the River Trent. It is located in Nottingham, the UK. Studies were carried out to estimate its dynamic properties using smartphone cameras [80] and GNSS [114,115]. In this study, a video dataset from a smartphone, in which vibrations of the bridge are induced by the synchronized jumping of students at the center of its midspan, is analyzed. The aim is to estimate the first vertical vibration frequency and its corresponding mode shape of the bridge. Figure 8 shows the bridge and locations of the CV-based measurement system. A 4k 30 fps video recorded with Smartphone S1 (Samsung Galaxy S9+) is used. It can be noticed that three more devices (one GoPro, and two more smartphones: Smartphone S2 and S3) are positioned near the bridge. For brevity, observations from these measurements are omitted in this case study. The field of view (FoV) of S1 is very similar to the photo showing camera locations in Figure 8.

**Figure 9:**
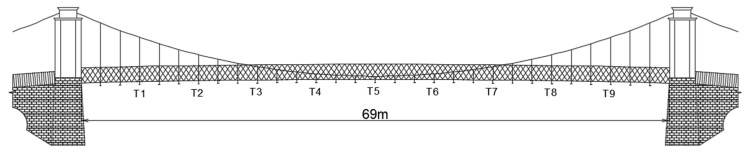
A sketch of the Wilford Suspension bridge with the locations of targets (Ti, where i=1, 2, …, 9).

**Figure 10:**
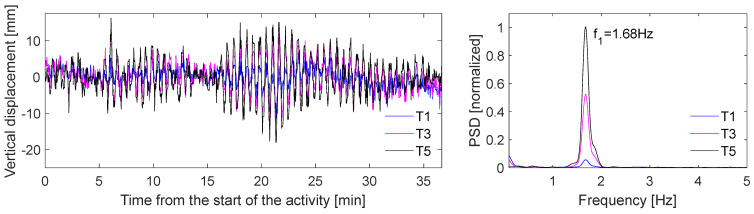
Vertical displacement time-history for the selected activity (**left**) and power spectrum density plot (**right**).

**Figure 11:**
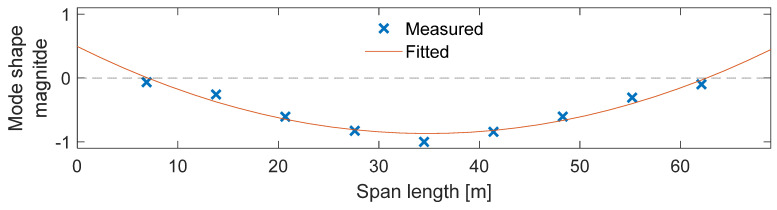
The mode shape of the bridge at its first vertical frequency.

Figure 9 shows the location of selected targets, which are hanger connections to crossbeams. The DIC technique is used to estimate target motions. Motions of a background target, which is a part of the building behind the bridge, are also collected to remove possible camera movements. Pixel displacements are converted to engineering units using the planar homography method, in which coordinates of four known points (i.e., two hanger connections to the main cable and crossbeams) are used as fixed and moving points. Time-histories of the vertical displacement of T1, T3, and T5 from Smartphone S1 are shown in Figure 10 (left). T5 is located at the mid-span of the bridge. It has the largest range of displacements. The Welch transform is selected to generate power spectrum density (PSD) plots (see Figure 10 (right)). The frequency signals are normalized setting the maximum value of T5 as 1. The first vertical frequency of the bridge is estimated to be 1.68 Hz, which is in agreement with findings from studies where GNSS measurements were used [114]. The PSD plot shows that the amplitudes of targets increase as their distance from the supports grows. Figure 11 confirms this. It shows the computed mode shape from target amplitudes. The mode shape resembles an anticipated shape derived from a combination of free-free and simply supported beams.

### 6.2. Estimating Bridge Vibration Parameters: A Case Study Utilizing Accelerometer Data

**Figure 12:**
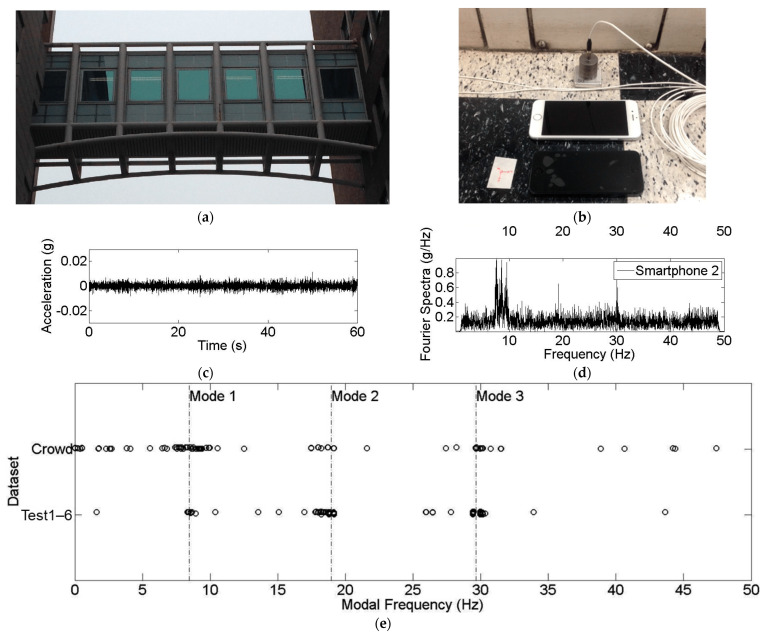
(**a**) Mudd–Schapiro Bridge, (**b**) accelerometer instrumentation, (**c**) sample smartphone measurement, (**d**) its frequency spectrum, and (**e**) and the comparison of crowdsourcing-identified modal frequency values with reference tests [34].

One of the benchmark testbeds Ozer [21] used was the Mudd–Schapiro Bridge (Figure 12a), an 11 m-long pedestrian link bridge connecting two high-rise buildings on the Columbia University campus. The Mudd–Schapiro Bridge hosted a series of concept-proof tests concerning citizen-induced smartphone sensor inaccuracies and their associated remedies, and this case study presents key features from those tests (e.g., Figure 12b presents phones placed on the bridge deck together with a reference piezoelectric accelerometer). The SHM approach in these studies were vibration-based; in other words, the methodology relied on obtaining accelerometer data from the bridge deck and conducting modal identification at various levels, some of which contain damage-sensitive features. The inception of citizen-engaged uncertainty formulations arose from a first-round of real pedestrian data collected from participants as crowdsourcers in [34]. Figure 12c,d shows an example signal obtained from smartphone submissions, particularly Smartphone 2, which corresponds to an iPhone 5. It was found that single-output data simply constituting Fast Fourier Transform outputs were scattered around the actual modal frequencies, yet with a significant dispersion, such as shown in Figure 12e.

Post-test conversations with the participants revealed that suggested instructions were not always strictly pursued (e.g., alignment vs. axis mismatch of the phone). This led to the development of potential error sources and proceeded with mitigation measures within the mobile sensing protocol. The authors identified three distinct citizen-induced features that can cause errors in the identification results. These were a (a) lack of control in the time and the location of the measurements, (b) distortions in the phone orientation, (c) and data corruption/losses due to the indirectness of the measurements, such as pedestrian-carried scenarios. To address (a), the authors proposed a signal-processing framework to handle spatial and temporal irregularities in the smartphone data [55]. Concerning feature (b), the authors proposed a coordinate system transformation procedure to retrieve the undistorted smartphone data and remove orientation errors [56]. Finally, feature (c) was addressed via a biomechanical model development process that understands the spectral features of the intermediary media (e.g., pedestrian) and removes them from the indirect smartphone data via an application of a transfer function [57]. Eventually, the authors discussed how the identification results can be further informed to go beyond modal identification and support digitalization processes (e.g., finite element model updating followed by reliability problems) [63].

**Figure 13:**
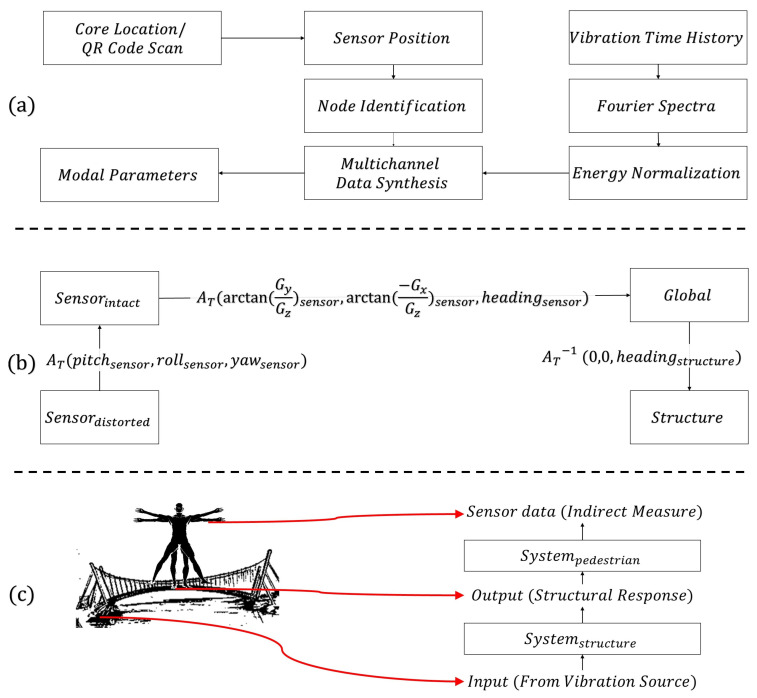
Smartphone-based SHM frameworks for (**a**) spatiotemporally uncontrolled [55] and (**b**) directionally distorted [56] and (**c**) and indirectly retrieved vibration data [57].

Figure 13 presents how each mitigation measure contributes to the accuracy of spectral features. In Figure 13a, the authors propose an energy-to-power conversion scheme for treating the spatiotemporally irregular vibration data collected from smartphones [55]. Another expected uncertainty due to the uncontrolled sensing environment is sensor orientation changes and errors. Figure 13b proposes a framework to recover distorted vibration signals in the correct alignment with the help of magnetometer, gyroscope, and accelerometer data fusion [56]. Finally, indirect sensing media are the standard concerns in smartphone-based SHM. Figure 13c addresses the indirectness problem using a transfer function development scheme that learns the vibratory features of intermediate bodies to subtract them from the indirect signals and retrieve bridge features [57]. Finally, Table 3 classifies a few benchmark metrics showing the identification challenges addressed in the studies, main impacts, and signature problems that need further attention to achieve improvements in the results.

## 7. Emerging Matters, Challenges, and Discussions

The techniques discussed above and the LoM formulation classify the majority of the smartphone-based bridge monitoring studies into three segments known to the authors. However, cases that (i) may not fully agree with the description of those LoMs and (ii) may use a radically different sensor technology exist. In this section, we briefly introduce those matters, some of which are underrepresented in the literature and identify the future steps in smartphone-based bridge monitoring research.

### 7.1. Load Identification

Getting started with the themes that partially fit the above descriptions, one key matter is load identification, rather than structural response measurements. The bulk load of the literature on smartphone-based SHM typically compares the identification results with a reference sensor network or compares the measurements with reference sensor technology. These are relevant to the structural response; however, bridge safety relies on the collocated statistics of demand and capacity, where little attention was paid to the former. A few studies presented findings relevant to load identification, for example, smartphone-based load estimation was proposed by Chen et al. [144], as well as the last phase of the Mudd–Schapiro case study introduced in the previous section [57]. Mustapha et al. took it to a further knowledge level with a data-driven identification approach, utilizing training datasets to understand pedestrian loads gathered from multisensory smartphone data [145]. It is essential to spend more effort on load identification using smartphone data, because the knowledge of loads does not only give the administrators an opportunity for capacity comparisons but also converts the identification problem from output-only to input-output approaches where more knowledge is available (and fewer assumptions are made, accordingly).

### 7.2. Unconventional Sensor Technologies

A dominant group of research conducted by the smartphone-based SHM community relied on vibration-based techniques with the particular usage of either accelerometers or computer vision (in some cases both). Sensor fusion did include data apart from accelerometric measurements; however, in most cases, these datasets had supplementary functionality to support the betterment of the acceleration response (e.g., [56]) obtained from accelerometers or displacement data obtained from smartphone camera/lens configurations. There are few studies that employed outside-the-box techniques where neither the accelerometer nor the camera/lens was the main tool adopted in the study, e.g., Nazar et al. [146] detecting the cracks on steel plates based on magnetometer readings with a contact-based SHM solution. Oraczewski et al. [67] developed another unusual approach that integrated a smartphone with a transducer performing the nonlinear acoustics-based crack detection of plates. Nonconventional approaches are not limited to invasive smartphone-based SHM techniques. Another interesting example is a non-contact approach that utilizes Light Detection and Ranging (LIDAR) technology embedded in the front face of new-generation smartphones for modal identification [66]. With more types of sensors embedded in smartphones, improvements in sensor quality, and exponentially increasing on-board intelligence, we expect to observe more innovative approaches that come up with completely new implementations or identification/detection theories involving smartphone data. More experimentation is needed to discover the creative sensing environment supported by many other sensing approaches, e.g., ambient light sensor [147] or audio classification [148].

### 7.3. Reaching Ubiquity in the Infrastructure Scales and Disruptive Paradigms

When smartphones first emerged as a future infrastructure monitoring paradigm, the key selling notion was the unprecedented scales of measurements and datasets that could have been achieved with billions of devices and big data behind it, as Feng et al. noted [33]. Most of the studies were retained within single-digit structural testbeds, except a few [41,42]. However, very few studies went beyond proof-of-concept and achieved real crowdsourcing findings, and they did not address a large population of infrastructure. A few exceptions to crowdsourcing data were [34] in a stationary, yet uncontrolled, sensing environment, whereas [131] presented drive-by modal identification evidence obtained from phones located on moving vehicles, again including uncontrolled datasets. Incentive is one core characteristic that is yet to be discussed thoroughly to understand how crowdsourcing can be motivated; however, perhaps due to the qualitative notion of the term, there is little-to-no development in this direction. Perhaps sharing data and ethical constraints associated with it hinder its further development. There are interesting examples in bridge engineering research, e.g., gamified platforms supporting inspection processes [149] that could also bind well with the smartphone-based approaches or advances in human-computer interaction that can take the crowdsourcing experience to a different level [150]. In our opinion, crowdsourcing requires a transformative approach where most of the sensor technologies are not known by society. With the right incentives, public participants can be trained to take on a role in post-disaster scenarios and inspect infrastructure conditions at regional levels [151]. Experimentation and learning via smartphone sensor technologies necessitate a structured educational approach, and its role can reshape the civil engineering curriculum, while preparing tomorrow’s crowdsourcers.

### 7.4. Reflection on LoM Contributions from the Literature and Observable Trends

The majority of the bridge monitoring (or a few of their transferrable counterparts) studies incorporating smartphones fall into the three LoM schemes. While the authors do not attempt to have an exhaustive list of all possible publications relevant to these schemes, the statistical observations from this literature provide insight into how the SHM community is adopting smartphone technologies. The following conditions are set when the contributions to statistics are accounted for: (I) only smartphone-related bridge SHM contributions are regarded, in other words, non-bridge applications are predominantly disregarded (e.g., building and pavement monitoring) unless there is a directly transferrable technique; (II) only journal articles are considered, avoiding overlaps and relying on rigorous review processes (III) official volume years are tagged (rather than the online publication date). In addition to these, some papers might overlap in multiple LoMs depending on their context.

**Figure 14:**
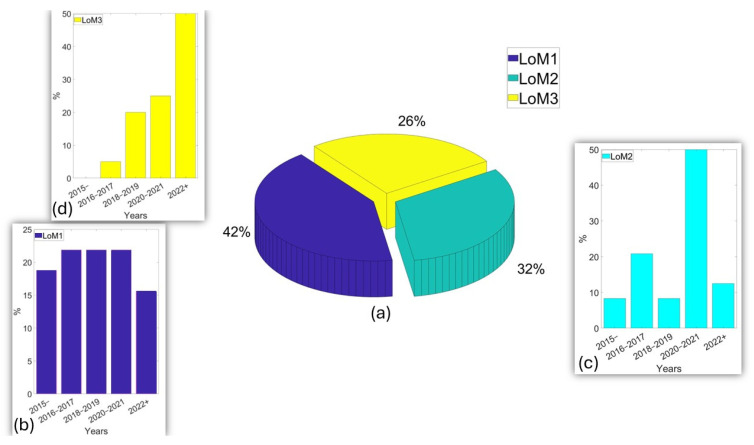
(**a**) Overall distribution of smartphone-based bridge monitoring sample studies according to their LoM context; (**b**–**d**) yearly publication trends among LoM1, LoM2, and LoM3, respectively.

A sample set of 76 is achieved. The set could be further extended if considering non-bridge infrastructure, and their distributions among LoMs and temporal trends are noted. As a reminder, LoM1 corresponds to contact and stationary accelerometer readings, e.g., [152,153], LoM2 represents noncontact and vision-based readings, e.g., [154], and LoM3 refers to drive-by or vehicular bridge monitoring (e.g., [37]) and arguably can also take different indirect forms, such as pedestrian-carried sensors, e.g., [155]. Figure 14 presents four relevant metrics. The first metric (Figure 14a) shows that the journal paper population in hand suggests 42% of contributions in LoM1, followed by approximately 32% and 26% of contributions for LoM2 and LoM3, respectively.

The contribution tallies are as expected since LoM1 is the closest smartphone-based SHM domain to traditional vibration-based SHM, which has been in place for more than four decades, e.g., [156,157]. Compared with that, computer vision-based displacement monitoring backing LoM2 had the first solid applications around 1990s [158], which was introduced relatively late in the SHM literature. However, the drive-by SHM—and accordingly, LoM3 principles—was introduced around 2005 [159]. There is a size difference among the communities involved in SHM research domains. LoM3 is the most niche area, whereas LoM1 can be adopted by the broadest SHM community possible. Other than that, Figure 14b–d presents the yearly contributions to each LoM, and there are noteworthy differences. In LoM1, there is a steady trend towards the years (ranging between 15% to 25% in the literature per two to three calendar years), whereas LoM2 shows more fluctuations in yearly contribution numbers. And most importantly, LoM3 started relatively late but has an observable momentum with the radically increasing number of publications in the last few years (i.e., 50% of the contributions were published between 2020 and 2022). The authors expect that LoM3 research will dominate the forthcoming decade in smartphone-based SHM literature, since it is the most mobile, crowd-friendly, and resilient SHM approach, despite the challenges observed in terms of short-sampling durations and the indirectness of the measurements.

### 7.5. Future Directions and Vision of Smartphone Enabled SHM of Bridges

In summary, the smartphone-based monitoring of bridge vibrations and deformations is interdisciplinary and necessitates a balanced representation of engineering mechanics and computer science fields. Detailed reviews targeting various aspects discussed herein show the diversity of the technical background that supports this line of research. For a broader scope of smart SHM beyond a sole smartphone focus, the readers are referred to [43]. Another review looks at different sensor technologies embedded in smartphones [160] and another review presents a literature analysis investigating mobile crowdsourcing in a general sense [161]. SHM field has been under the impact of machine learning advances and rapid developments in the intelligence of the monitored assets [46], e.g., advancements in deep learning [162] and pattern-recognition problems in a more comprehensive manner [163], which are likely to impact smartphone-based bridge monitoring research as well. Finally, this review particularly focuses on the bridge SHM domain and proposes the associated LoM concepts per each distinct approach. On the other hand, smartphone-based SHM research gets richer as the scope is expanded to other infrastructure. And for those with further interest in other SHM domains, we recommend other review articles with a broader civil infrastructure scope (e.g., [44]).

**Figure 15:**
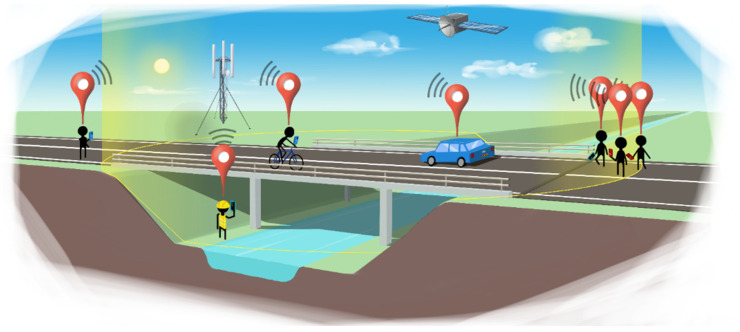
A vision of collective and connected smartphone applications for bridge SHM.

To summarize, the authors envision an integrated and synergistic platform where smartphone data sources are not isolated from each other. There is a correlation in many sensory readings, as well as their relationship to structural conditions; why restrict ourselves to one subset of smartphone technology? While each technology has its own heterogeneity, different metrics can learn from each other to generate a strong damage indicator from a bridge-monitoring point of view. A few examples are shown in Figure 15: handheld devices, pedestrian data, crowd data, micromobility or traditional vehicular data, non-contact vision-based data, IoT (such as weather data), and more techniques will arise as new sensors are introduced in the smartphone industry. It would be vital if some of the producers started to consider the need for an ambient civil infrastructure monitoring paradigm that can run these data feeds in the background, because smartphones have proven their capability to produce digital knowledge that could have only been produced by scientific instrumentation that was not accessible to the public in the near past.

A few final recommendations to capture smartphone-based ubiquitous bridge data in the future rely on delving into data ownership and security/privacy matters. While vibration data at a first glance may not appear as a privacy-sensitive matter, supplementary datasets supporting the signal processing phases may indicate otherwise (e.g., location services). This requires a further exploration of ethics and governance, which may not be largely discussed by the SHM community in the past. And incentives—mentioned earlier—rely on public and industrial collaborations. Marketing campaigns supported by transportation authorities can inform the public of the importance of smartphones as near-scientific instruments and let them know that their data can make a difference. Likewise, asset or service owners (e.g., delivery services and transportation hubs) involved in vehicular networks or smartphone populations can take the lead to add value to the idle sensors in smartphones waiting to function for better infrastructures.

## 8. Conclusions, Vision, and Future Opportunities/Directions

Advances in computation and electronics have brought affordable mobile devices equipped with a deluge of sensors and components making them ubiquitous in the modern world. The initial purpose of mobile phones, which was to make calls and send messages, has changed making them smartphones. Modern smartphones are used for many purposes for which they were not initially built. In this review, we have researched the available literature on smartphone applications in bridge structural health monitoring (SHM). Early studies have shown the applicability, precision, and resolution of smartphone vibration measurements with a diverse range of testbeds from all around the world. Evidence shows that there is an application potential, especially since civil infrastructure (including bridges) is within an identifiable range of dynamic character (i.e., <20 Hz). The review followed the tradition to introduce the commonly used smartphone built-in sensors for bridge monitoring, studied their applications, and then introduced a new classification concept according to the Level of Mobility (LoM) to characterize the mainstream research directions taking place mostly in the last decade.

Three LoMs are identified to define the bulk population of SHM research using smartphones, shortly: 1-invasive/dedicated, 2-noncontact, and 3-vehicular/mobile. Their spatial footprints on civil infrastructure, such as bridges, are tremendously different. That is to say, LoM1 samples can be quite long and relatively accurate, yet with discrete sensor location(s). LoM2 relies on camera/lens configurations and can be deemed the same in terms of the sampling length. Due to the nature of the physical parameter being observed, LoM2 may or may not be applicable for dynamic characterization; however, in some cases, it might be used to monitor static deformations and its products (displacements, strains, and so on). And LoM2 can monitor a subset of the infrastructure in a continuous and spatially distributed manner as long as the region of interest is within the frame.

The final class is LoM3, which has the highest spatial resolution, yet is subject to practical challenges such as distortion of the signal due to intermediary media (e.g., vehicle mechanics), as well as super-short samples acquired during vehicle passage. While it can be argued that data will self-cleanse in the long run and damage-sensitive feature patterns will become visible as the dataset grows, it is a well-known fact in the SHM community that the reality suggests differently. Scientific instrumentation, let alone smartphones, has limitations in identifying damage in complex and bespoke structural assets, where material characteristics and production processes account for high levels of uncertainties and safety factors. Some of these uncertainties are not irreducible with large numbers of samples, and others can lead to false alerts due to non-damaging effects, e.g., environmental and operational variability.

Nevertheless, interest in smartphones and their offered technologies has gradually increased within the last decade despite their numerous challenges, such as low-fidelity specifications, clock imperfections, synchronization errors, and an uncontrolled measurement environment. Current literature is still dominated by technology verification, but the first examples from large-scale and real-life cases are finally on the horizon. The next decade will show more data-centric research incorporating data batches and more physics-informed approaches merging digital twin theories with smartphone data. Other sources of intelligence will provide further support in the process: it is the Internet of Things age where smartphones are connected to everywhere and can be trained node-by-node or as a network (and/or can work as alarm triggers in individual or network scales) for a variety of different SHM problems.

## Figures and Tables

**Table 1:** Summary of the LoMs and key characteristics.

Characteristics	LoM1	LoM2	LoM3
Coupling Condition	Invasive	Noncontact	Indirect and Mobile
Key Sensing Feature	Acceleration	Displacement	Acceleration
Device Presence	Dedicated and On-Site	Dedicated and Remote	Nondedicated and Vehicular
Spatial Footprint	Low (Pointwise)	Medium (Multi-Point)	High (Span-wide)
Potential Size Per Sample	High (Minutes to Hours)	High (Minutes to Hours)	Low (Seconds)
Access at Population-Level	Limited (Requires incentive and labor)	Limited (Requires incentive and labor)	Yes

**Table 2:** Identification accuracies exemplified from LoM1 to LoM3.

Year/Source	Bridge Study Features	Smartphone Freq. (Hz)	Reference Freq. (Hz)	Accuracy Range	Clock Correction
2015/[33]	Multi-span prestressed concrete (LoM1)	3.16–3.03	3.13–3.00	0.96–1.00%	No
2015/[34]	Single-span metal frame arch (LoM1)	8.57–8.40	8.48	1.30–0.71%	No
2015/[49]	Stay cable frequency differences (LoM1)	3.70	3.68	0.54%	No
2018/[37]	Laboratory-scale simply supported beam (LoM3)	3.71	Not reported (seems identical)	Not reported (seems near 0)	Yes
2019/[83]	Single-span laboratory beam (LoM2)	30.59	30.64	0.2%	N/A
2019/[122]	Prestressed concrete with a series of simply supported spans (LoM3)	7.01–7.69	7.56	7.3–1.7%	No
2020/[77]	Multi-span concrete box girder (LoM1)	1.47–1.37	1.47–1.39	0–1.6%	Yes
2020/[59]	Long-span suspension (LoM1)	0.106	0.106	0%	Yes
2022/[73]	Multi-span concrete arch box-girder (LoM1)	1.51	1.47	2.4%	Yes
2022/[130]	Tied arch cable-stay (LoM3)	2.53	2.52	0.4%	Not reported
2022/[131]	Long-span suspension (LoM3)	0.106–0.108	0.106	0–1.9%	Yes

**Table 3:** Mobility specifications pertaining to Mudd–Schapiro tests.

	Reference [55]	Reference [56]	Reference [57]
Spatial Features	Location	Orientation	Moving or standing still
Temporal Features	Normalized with respect to sample size	Not addressed	Not addressed
Identification Scope	Modal characteristics	Modal characteristics	Modal characteristics
Contact Features	Direct (yet subject to stationary location change)	Direct (yet distorted)	Indirect (pedestrian carried)
Key Achievement	Can retrieve mode shapes from the irregularly positioned smartphone’s single-output data	Orientation distortions are identifiable, trackable, and reversible in the monitoring process	Can remove biomechanical effects from pedestrian data for bridge monitoring
Main Limitation	Near-zero modal displacements are largely impacted by the noise floor of the low-fidelity sensor and depends on the phone make/model	Magnetometer is very sensitive to the environment (e.g., metallic objects) that can adversely impact correction accuracy	Walk-induced vibrations are too dominated by the walking action itself and therefore mask bridge characteristics

## Data Availability

The data that support the findings of this study are available upon reasonable request.
